# Alopecia areata-like and psoriasis after dupilumab use for atopic dermatitis^☆^^☆☆^

**DOI:** 10.1016/j.abd.2021.03.005

**Published:** 2021-07-14

**Authors:** Viviane Maria Maiolini, Nathalie Andrade Sousa, Paula Figueiredo de Marsillac, Aline Lopes Bressan

**Affiliations:** Service of Dermatology, Faculty of Medical Sciences, Hospital Universitário Pedro Ernesto, Universidade do Estado do Rio de Janeiro, Rio de Janeiro, RJ, Brazil

Dear Editor,

Atopic dermatitis (AD) and psoriasis are chronic inflammatory dermatoses, considered to be extreme phenotypes of the immune mechanism modulated by T cells.^1^ Dupilumab inhibits the signaling of IL-4 and IL-13, cytokines that mediate the skin barrier inflammation and dysfunction in AD, through Th2. There is no direct interference on the Th1 pathway.^2^

The present report describes a rare manifestation of alopecia areata-like psoriasis, associated with skin lesions after dupilumab use.

A 22-year-old male patient was diagnosed with AD since childhood and submitted to several previous treatments, such as topical and systemic steroids, azathioprine and methotrexate, which were withdrawn due to therapeutic failure. Additionally, he had been prescribed cyclosporine, with a reduction in SCORAD (Severity Scoring of Atopic Dermatitis) from 58 to 18, but with dose reduction due to an increase in liver transaminases (3-fold the reference value). In this context, dupilumab was started at the dose recommended in the leaflet, subcutaneously. After five months, he developed a scaling, erythematous alopecia plaque, with pruritus, on the vertex region, measuring 5 cm in diameter (Fig. 1), with erythema and follicular and interfollicular desquamation, suggesting an eczema pattern on dermoscopy. Scaling erythematous plaques were observed on the left wrist and toes, as well as onychodystrophy on the right hallux and nail pitting in the fingers (Fig. 2). The direct mycological test and cultures of the alopecia plaque were negative. The histopathological analysis showed psoriasiform dermatitis with confluent parakeratosis, spongiosis, exocytosis of lymphocytes and extravasated red blood cells. PAS and Grocott staining were negative. Due to the probable diagnosis of psoriasis, topical betamethasone dipropionate and calcipotriol were initiated. After one month, the patient returned with areas of hair regrowth on dermoscopy and improvement of the skin lesions. After about nine months (SCORAD = 10.6), he showed complete hair regrowth and regression of the lesions (Fig. 3).

**Figure 1:**
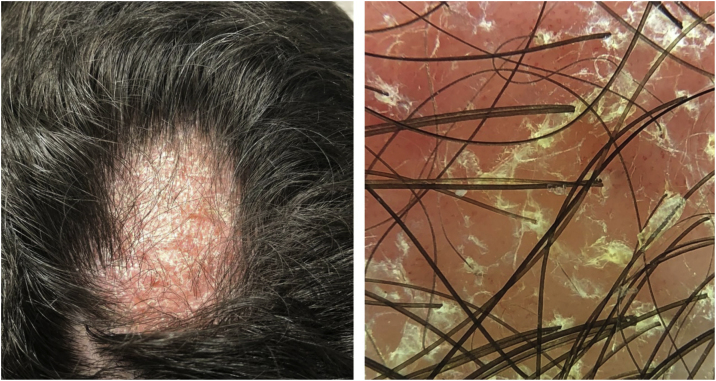
Erythematous-scaling alopecia plaque on the vertex region, measuring approximately 5 cm in its largest diameter. Dermoscopy showed erythema and follicular and interfollicular desquamation, suggesting an eczema pattern.

**Figure 2:**
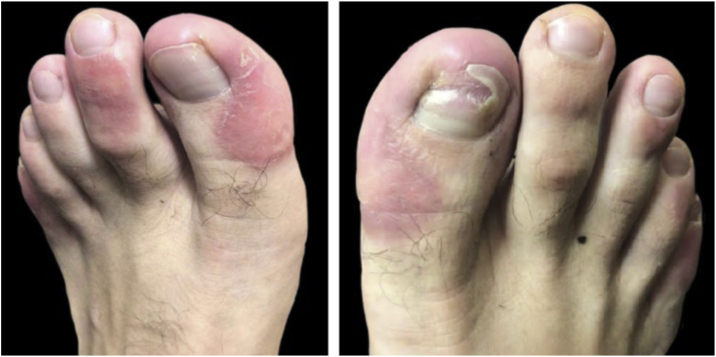
Erythematous scaling plaques on toes and onychodystrophy on the right hallux.

**Figure 3:**
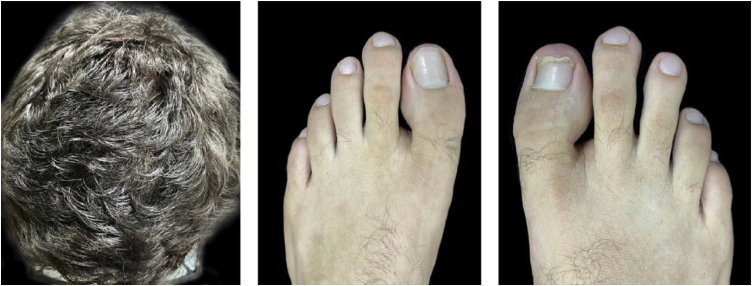
Ten months after the onset of the condition, with complete hair regrowth and remission of erythematous-scaling lesions on toes and onychodystrophy undergoing resolution on the right hallux.

AD and psoriasis are T-cell-mediated dermatoses.^1^ Th2 cytokines, such as interleukins IL-4, IL-13, IL-22, and IL-31 are implicated in the pathogenesis of AD.^3^ Th1 cytokines, such as IL-17 and IL-23 are implicated in psoriasis.^1^

Dupilumab, a fully human monoclonal antibody, blocks the α-subunit of the IL-4 receptor, which leads to the blocking of Th2 cytokine signaling (IL-4 and IL-13). There is a hypothesis that the antagonism of the Th2 pathway with dupilumab would lead to the opposite activation of the Th1 pathway. On the other hand, patients with psoriasis or another Th1-mediated disease could develop AD after treatment with immunobiologicals.^2^

There have been reports of alopecia areata associated with dupilumab use, as well as resolution or significant improvement of the pre-existing condition after dupilumab use.^4^ In the described case, there was an erythematous alopecia plaque, with interfollicular and perifollicular scaling on dermoscopy, suggestive of psoriasis, eczema, or *tinea capitis*.

Erythrodermic, plaque, acral, and scalp psoriasis have been described, but there is no description of psoriatic alopecia plaques (alopecia areata-like) associated with the use of dupilumab.^1^, ^4^ Alopecia is rarely directly related to psoriasis, with telogen effluvium being its most common form. In most cases, hair regrowth occurs, rarely progressing to the cicatricial form.^5^

There have been reports of psoriasis as an adverse effect of dupilumab, but it has not been included as an adverse event in clinical trials.^1^, ^4^ This case describes an unprecedented event associated with the use of dupilumab, which is important for the understanding and management of this drug.

## Financial support

None declared.

## Authors' contributions

Viviane Maria Maiolini: Drafting and editing of the manuscript; intellectual participation in the propaedeutic and/or therapeutic conduct of the studied cases; critical review of the literature; approval of the final version of the manuscript; critical review of the manuscript.

Nathalie Andrade Sousa: Drafting and editing of the manuscript; intellectual participation in the propaedeutics and/or therapeutic conduct of the studied cases; critical review of the literature; approval of the final version of the manuscript; critical review of the manuscript.

Paula Figueiredo de Marsillac: Drafting and editing of the manuscript; intellectual participation in the propaedeutics and/or therapeutic conduct of the studied cases; critical review of the literature; approval of the final version of the manuscript; critical review of the manuscript.

Aline Lopes Bressan: Design and planning of the study; writing and editing of the manuscript; intellectual participation in the propaedeutics and/or therapeutic conduct of the studied cases; critical review of the literature; approval of the final version of the manuscript; critical review of the manuscript.

## Conflicts of interest

None declared.
